# Enhancing primary care support for informal carers: A scoping study with professional stakeholders

**DOI:** 10.1111/hsc.12898

**Published:** 2019-11-26

**Authors:** Michele Peters, Stacey Rand, Ray Fitzpatrick

**Affiliations:** ^1^ Health Services Research Unit Nuffield Department of Population Health University of Oxford Oxford UK; ^2^ Personal Social Services Research Unit School of Social Policy, Sociology and Social Research University of Kent Canterbury UK

**Keywords:** carers, health and care services, primary care, professional stakeholders, qualitative

## Abstract

Informal carers (i.e. people who provide unpaid care to family and/or friends) are crucial in supporting people with long‐term conditions. Caring negatively impacts on carers’ health and experiences of health services. Internationally and nationally, policies, legislation, professional guidance and research advocate for health and care services to do more to support carers. This study explored the views of health and social care providers, commissioners and policy makers about the role and scope for strengthening health service support for carers. Twenty‐four semi‐structured interviews, with 25 participants were conducted, audio‐recorded, transcribed verbatim and analysed by thematic analysis. Three main themes emerged: (a) identifying carers, (b) carer support, and (c) assessing and addressing carer needs. Primary care, and other services, were seen as not doing enough for carers but having an important role in identifying and supporting carers. Two issues with carer identification were described, first people not self‐identifying as carers and second most services not being proactive in identifying carers. Participants thought that carer needs should be supported by primary care in collaboration with other health services, social care and the voluntary sector. Concerns were raised about primary care, which is under enormous strain, being asked to take on yet another task. There was a clear message that it was only useful to involve primary care in identifying carers and their needs, if benefit could be achieved through direct benefits such as better provision of support to the carer or indirect benefit such as better recognition of the carer role. This study highlights that more could be done to address carers’ needs through primary care in close collaboration with other health and care services. The findings indicate the need for pilots and experiments to develop the evidence base. Given the crucial importance of carers, such studies should be a high priority.

**What is already known about this topic**

- Carers provide crucial support to people with long‐term conditions but frequently are not supported themselves
- Carer policy advocates greater support for carers but it is unclear how this has been translated into practice
- There may be a significant gap between aspirations to strengthen the role of primary care in supporting carers and real‐world capacity to deliver this support

**What does this paper add**

- Professional stakeholders agree that primary care, in collaboration with wider health and care services, have an important role in identifying and supporting carers
- Currently primary care, and other services, are not proactive enough in identifying and supporting carers
- There was consensus that processes for identifying carers and their needs were only useful, if benefit to carers is achieved

## 1 INTRODUCTION

Informal or unpaid carers play a crucial role in providing care and support for individuals with health problems. Informal or unpaid carers play a crucial role in providing care and support for individuals with health problems. An informal carer is someone who provides unpaid help and support to a partner, child, relative, friend or neighbour who could not manage without this help (Beesley, 2006). Being a carer is associated with poorer mental and physical health (Hiel et al., 2015; Peters, Jenkinson, Doll, Playford, & Fitzpatrick, 2013; Thomas, Saunders, Roland, & Paddison, 2015) and poorer experiences of using primary care (Thomas et al., 2015). A study of carers in England found that people who choose to be carers had better quality of life and less carer strain than people who provided care as it was expected of them (Rand, Malley, & Forder, 2019). Poorer experiences of services are associated with poorer carer quality of life (Peters et al., 2013). The most pressing needs of carers are information and training, professional support, effective communication (with the person they care for but also with professionals) and financial and legal support (Silva, Teixeira, Teixeira, & Freitas, 2013). Two‐thirds of Australian carers report unmet need including needs for financial, physical and emotional support (Temple & Dow, 2018). In the UK, carers believe that health professionals may be in a unique position to validate their role as a carer and to signpost them to support (Knowles et al., 2016).

Poorer health‐related quality of life and experiences of services of carers mean that globally more needs to be done to support carers (Sheets, Black, & Kaye, 2014). Therefore policies, legislation, professional guidance and research all emphasise the case for identifying carers and addressing their needs (Bruening et al., 2019; National Health Service (NHS), 2019; NHS England, 2016; The Stationary Office, 2014; Watkins, Rimmer, & Muir, 2013). In Europe, support policies for carers vary. Despite views that supporting carers is of equal importance to supporting the person they care for, most countries do not have a mechanism for identifying carers and assessing their needs (Courtin, Jemiai, & Mossialos, 2014). Policies that provide carers with free time, support carers emotionally and give them skills to improve the care situation are associated with better outcomes (Calvo‐Perxas et al., 2018).

Recognition by policy and research of the vital contribution of carers is one factor stimulating health services, including for example the National Health Service (NHS) (NHS England, 2016) and US health services (Bruening et al., 2019), to focus on supporting carers. Despite clear recognition of the importance of health services supporting carers, in England only a fraction of the healthcare budget is spent on carers (Morgan, 2016). Furthermore, the nature of health services’ contribution to carer support is still not well defined. For example staff in primary care are uncertain as to their role and believe that they lack time, resources and skills to play a more extensive role in supporting carers (Greenwood, Mackenzie, Habibi, Atkins, & Jones, 2010; Silva et al., 2013; Simon & Kendrick, 2001).

Despite current intentions to progress towards the integration of care, in England, the main responsibility of supporting carers still rests with social care, delivered by local authorities (LAs) (public bodies setting local priorities). The NHS is a centralised, free at point of delivery service whereas social care, which is administered by LAs, is local and means tested. The NHS provides healthcare whereas social care, provided by LAs, offer a range of practical support such as day centres, equipment, meals, home care and nursing homes). LAs can partly fund the voluntary sector (i.e. carer charities) to help address carer needs. The Care Act (The Stationary Office, 2014) is a clear legal framework and places responsibility of addressing carer needs and quality of life on LAs. Implementation towards integrated care is hindered by a lack of continuity and coordination of care and limited involvement of service users and their carers in care decisions (Sadler et al., 2019).

There may be a significant gap between formal policy aspirations to strengthen the role of health services in identifying and supporting carers and real‐world capacity. A scoping study was undertaken to explore the views of professional stakeholders on how health services, in particular primary care, can support carers and scope for strengthening such support in England.

## 2 METHODS

In this scoping study, qualitative semi‐structured interviews were conducted with professional stakeholders (policy makers, commissioners, front line clinicians, LA staff and voluntary sector organisations) between December 2016 and March 2017. The Central University Research Ethics Committee, University of Oxford, classified this project as a pre‐research activity (2 November 2016) and advised that no formal ethical approval was necessary.

A scoping review was conducted on health and care policy specific to carers, and research on carer needs, outcomes and support from services. The semi‐structured topic guide was developed by the authors on the basis of this review and their expertise on carer research. The topic guide was tested for its content in a mock interview. Questions focussed on the participant's role and interest in carer‐related issues; the role of primary care and other health services in identifying and supporting carers; and the potential usefulness of a carer assessment tool within health care settings.

### 2.1 Participant recruitment

Potential participants were invited through a combination of convenience and snowball sampling by two interviewers (Author 2 and research assistant). Author 2 is an experienced qualitative researcher and the research assistant was trained by Authors 1 and 3 (experienced qualitative researchers) for the purposes of this study. Stakeholders, with an interest in carers of someone with a long‐term chronic condition, known to the researchers (as they had been participants of or collaborators on previous studies or part of the network of the researchers) or with publicly available contact details were contacted via email.

The intention was to recruit a mixed, but pragmatic, sample of policy makers, commissioners, front line clinicians, LA staff and voluntary sector organisations. A total of 54 stakeholders were contacted; no response was received from 19, nine responded that they were unable to participate (usually due to time constraints) and one email was undeliverable. The majority of invited stakeholders were front line clinicians (predominantly GPs, but also nurses or some specialist doctors) from different parts of the England. A wide range of Voluntary Sector Organisations were contacted, including some generic (such as Age UK) and some disease specific (such as Macmillan Cancer, the mental health charity MIND). Local carer organisations across England were also invited. In terms of policymakers or commissioners, mostly NHS England staff were invited, but invitations also included the Care Quality Commission, one local CCG, two local councils and one LA. Stakeholders who participated in an interview, were asked for suggestions of other stakeholders to invite (snowball sampling).

Interviews were conducted by telephone unless the participant preferred a face to face interview. All interviews were arranged at the participants’ convenience. The majority of interviews were conducted over the telephone (*n* = 21); for the face to face interviews (*n* = 3), the researcher travelled to the participants’ place of choice (usually their place of work). Interviews were audio‐recorded, following informed consent which was taken verbally by the interviewer, and transcribed verbatim by a professional transcriber. The transcripts were checked by the research assistant against the recordings and any necessary amendments were made. Interviews lasted on average 42 min (range 22–66 min). Data collection continued until thematic saturation (i.e. no new themes were identified [Fusch & Ness, 2015]) was achieved.

### 2.2 Analysis

An inductive thematic analysis was undertaken. The analysis started in parallel with data collection to enable the findings from early interviews to shape subsequent interviews. A thematic framework was built from the analysis of six early interviews. Each author analysed six transcripts and developed their individual draft coding framework. The themes identified by each of the authors overlapped significantly and the three draft frameworks were brought together into one final framework during a meeting. The final framework was applied systematically to the analysis of all interviews by Author 1.

## 3 FINDINGS

### 3.1 Participants

Twenty‐four interviews, with 25 participants (two stakeholders participated in a joint interview) were conducted. Twenty‐one interviews were conducted by telephone and three face‐to‐face. Table 1 shows the role(s) held by participants. Five participants spontaneously volunteered the information that they have or used to have a carer role, with one stakeholder having provided support to three different family members.

**Table 1:** Participants’ role(s)

Role	*N* a
Front line clinicians	
GP	4
Nurse	4
Community pharmacist	2
Specialist/Consultant	1
Phlebotomist	1
Policy and commissioning	5
Voluntary sector	8
Local Authority/Social care	1
Private health care sector	3
Researcher	1

a Some participants had more than one role (current or past).

### 3.2 Themes

Three main themes were identified from the interviews: (a) identifying carers, (b) supporting carers and (c) assessing and addressing carer need. All participants agreed that the carer role is important, with some describing carers as ‘crucial’. There was a broad agreement that carers should be supported by primary care and other health and care services due to the impact of caring on the carer's own health and quality of life. It was acknowledged that without support some carers may not be able to continue caring. Although the participants agreed that primary care has a central role in identifying and supporting carers, their view was that primary care needs to work together with other health and care services to provide support to carers. The three themes are described in more depth below.

### 3.3 Identifying carers

Participants all thought it was important for services to identify carers as only if they are identified can support be offered. Generally, there seemed to be an issue with health services focusing enough on carers.

… the health service is bad at identifying and supporting carers and actually giving them status… carers are being undervalued, underappreciated and actually the sustainability of the NHS has been built on carers… what’s tending to happen is carers are not identified… healthcare professionals, I think, just focus on the patient and their job being to provide their clinical intervention. Whereas they are missing a trick, which is that wider support network of informal carers… (Participant 2, Policy and commissioning)

Participants identified a range of barriers in carer identification including people not self‐identifying as a carer, health services focusing on the person with the long‐term condition rather than the carer; carers being low on the healthcare agenda; not having a proactive mechanism for consistent carer identification; lack of understanding or knowledge of the impact of caring; and carer information not being shared due to IT systems not being linked. Even when a professional is aware of carers, there was a certain perceived ‘danger’ to asking the question.

… we’re faced with quite a resistance around people, that fear of opening a can of worms. So ‘if I ask them about being a carer then they’re going to want support and then I don’t know where that support is’, that kind of thinking… (Participant 22, Voluntary sector)

… we have a practice policy here to collect information about carers on an *ad hoc* basis. I have to say that what that means is it often doesn’t get done until you run into problems and you’re scraping around looking to find out who the carers or informal carers might be… (Participant 4, GP)

People not identifying themselves as carers was thought to be due to people thinking ‘carer’ means a paid care worker and seeing themselves primarily as the relative or friend of the person they care for. Furthermore, with the exception of an acute situation where someone can become a carer instantly, the caring situation develops slowly over time and carers may be unaware of the additional tasks they have taken on until they reach crisis point. Participants also acknowledged that the caring role evolves and changes—a carer may take on more caring tasks or if the person they care for dies, the caring role ends. Therefore, identifying carers was seen as an ongoing activity and efforts need to be made for that information about caring stays up to date. This adds further to the challenge of identifying carers.

… caring as a family member, you don’t see [it as] somebody putting more and more burden upon you because you just take it in your stride day after day, week after week. Which in other words, they’re edging towards carer stress and strain without knowing it … (Participant 8, Private healthcare sector)

Participants offered thoughts on potential solutions or mechanisms for identifying carers. Some thought that primary care could ask all people on their register if they are a carer whilst others believed that a targeted approach would be better, for example, by asking people with long‐term conditions, frailty or disabilities if they have a carer. Some examples of good practice were given such as primary care proactively asking their service users if they are a carer or having a carer register. However, it seems to be the exception rather than the rule. Most carers are identified opportunistically.

… one chap I saw a couple of weeks ago who, the only reason I realised that he had taken on a major carer role was his comments explaining why his home blood pressure readings were so much higher than the ones here. And I had said, ‘Why do you think that might be?’ and he said, ‘Oh, it’s probably the stress of looking after my wife,’ who, I hadn’t realised, had developed dementia… (Participant 15, GP)

… I suspect doing it on a formal basis, looking at disease registers and then proactively contacting people to find out who carers might be, try to get a list that way would be a much better way of doing it. We haven’t gone down that line, but I’m sure it is done much better elsewhere. But I think if you leave things on an ad hoc basis then it’s just one of the things that slips off the agenda, because often these patients are very complicated and there’s lots of things to think about… (Participant 4, GP)

### 3.4 Carer support

This theme focused on how primary care could or should be involved in supporting carers, in collaboration with other services, and reflected on barriers in delivering this support. A variety of reasons for supporting carers were highlighted such as preventing carers’ ill health or reaching ‘crisis point’; or ensuring effective support for the person cared for. Potential primary care roles included identifying carers; supporting them; providing reassurance, information, health checks and flu jabs; or signposting them to other services.

Although potential important roles of primary care are recognised, most participants believed that primary care (and health services generally) do not provide enough carer support. Despite some examples of good practice (such as some primary care practices having a carer champion; having carer registers in primary care; fast referral systems to social care; and cancer and dementia specialist services being more aware and supportive of carers), the predominant view was that there was wide variation in carer support provided by primary care and wider health services, with support for carers mostly left to social care.

… our colleagues in social care had gone absolutely hell for leather to try and make themselves Care Act compliant in the very short time between the law being passed and its implementation. And I think they did a phenomenal job actually… What came through very clearly though, because the NHS don’t have a legislative duty in the [Care] Act, they have a duty of cooperation, health had pretty much stepped away… (Stakeholder 10, Policy and Commissioning)

Primary care was seen as a crucial first point of contact, but participants stressed that a whole systems approach, integrating different services, is needed to effectively support carers. There was variation in how the ‘whole system’ was described but for the majority it included primary care, hospitals (including A&E services), LA social services and the voluntary sector. A small number of participants also included pharmacists, community services, dentists and care workers, as well as institutions beyond health and social care such as the police, fire departments, Parish Councils or faith groups. Within such an integrated system, all professionals would be expected to think about carers.

… We’ve done some work in partnership with the Fire Brigade over the last year or so, looking at, when they do their safe and well checks in people’s homes, they’ve actually managed to identify a lot of older carers by going into people’s homes. So actually there’s a lot about partnership working with different organisations and thinking about who is likely to have contact with different people… (Participant 10, Policy and commissioning)

Concerns were frequently raised about the current workload of GPs and primary care, about financial resources and a lack of knowledge on how to support carers or which other services to signpost them to. Some participants thought a major cultural change was needed as primary care focuses on treating ill health rather on providing carer support.

… the perception is GPs are just too busy to even do their primary function, providing the healthcare… the carer is not their patient, so it’s kind of not, almost not their business, not their concern… (Participant 22, Voluntary sector)

Participants emphasised that the resource strained primary care meant that change would be difficult to achieve. Whilst some of the voluntary sector participants highlighted initiatives to support primary care (as evidenced by the example below), there seemed to be a lack of leadership amongst policy makers and front line clinicians to take responsibility for driving change forward for carers. The voluntary sector though is limited to raising awareness and producing guidelines; initiatives of front line clinicians and managers of services are needed to drive change forward.

… I’m working with some colleagues, including GPs, to look at how we can raise awareness of the needs of carers amongst practice staff and GPs, and support primary care professionals to identify carers and make sure they get the support they need… what we’ve done in the past is develop guidance for health professionals on identifying carers… we’ve actually adapted that guidance specifically for primary care professionals… (Participant 20, Voluntary sector)

### 3.5 Assessing and addressing carers needs

The third theme focuses on participants’ views of implementing systems to assess carers’ needs. Currently, LAs are responsible to assess carer needs as legislated in the Care Act (The Stationary Office, 2014), however, carer assessments can be delegated to the voluntary sector. Broadly participants were supportive of a proactive mechanism for identifying and supporting carers. They thought that routine assessment of carers’ needs through primary care should be feasible. Many participants thought this identification and assessment could be achieved through a simple self‐completion questionnaire. There was strong agreement that an assessment, if it was implemented, needed to achieve benefit for the carer.

Participants identified two types of benefits: first, specific benefits such as receipt of helpful advice, information, respite services or treatments; and second, non‐specific benefits arising from recognition of the carer role. Participants were supportive of the assessment being used to identify unmet need that can consequently be supported or signposted to other appropriate services. Other benefits included the ability to contact carers when necessary; help for carers to support the person they care for, and the ability to inform carers about free flu jabs, health checks and in‐depth carer assessments. However, systems and services would need to be in place and health professionals need to be aware of these services to enable them to signpost carers to them.

…such a tool could be helpful for when people are bravely soldiering on and not realising – or not wanting to acknowledge the extreme pressure they are under. But of course, that only follows if we can follow that up with some truly meaningful support… (Participant 15, GP)

… if GPs are going to be signposting as a result of these assessments, it’s important that they have those links with the community and voluntary sector as well as social services… (Participant 20, Voluntary sector)

Organisational, cultural and structural barriers, and some potential solutions, in implementing a carer identification and needs assessment tool were described. Most participants pointed out that primary care is overstretched, so that it would be difficult to implement anything new. Therefore attempting to implement a carer tool needs to include mechanisms for minimising the burden using this tool and show true benefit. Participants thought it may be possible to achieve this by asking primary care solely identify carers and signpost them to other services for support. A potential cultural barrier was the view that LA social services are responsible for carers and therefore carers are not on the health services agenda. This may be resolved by providing training to health professionals. The main structural barrier was IT services not being allowed to share information with other organisations.

…if it’s going to take a lot more work to administer the survey then that’s going to be greeted with arms raised and lots of sighs. If then the information that they get takes a lot if inputting onto computer or it takes lots of dealing with the aftermath, if you suddenly find that you’ve got 50 carers saying they’re not coping and say they’re about to throw in the towel, kind of thing, then primary care are going to worry about that. I guess what you have to show is that there is real benefits from having this information to get people to engage with it… (Participant 4, GP)

Barriers, and potential solutions, were also identified from the carer perspective. First, it was thought to be difficult to identify and target the most appropriate population for a carer assessment. Challenges with identifying the target population were concerns about people not identifying themselves as a carer and the changing nature of the carer population, that is, people starting or stopping being a carer and the caring role evolving if the health of the cared for person deteriorates. To reach a maximum number of people, they supported a wide approach through a large range of health (e.g. primary care) and community settings (e.g. libraries, luncheon clubs, day centres) with different modes of administration (paper, online and smartphone applications).

…having a questionnaire is useful in that it would be useful to gain information from people about their caring roles. But it’s making sure it gets to the right people… (Participant 1, Nurse)

Participants had little, if any knowledge, about existing carer self‐reported questionnaires that may be used for identifying and supporting carers. Participants from the voluntary sector had either personally or through their organisation been involved in the use of carer instruments. Some of these were quite simple tools such as asking for the carer's contact details to enable the organisation to invite the carer for a meeting to discuss their needs. A few participants described existing carer self‐report instruments such as the Adult Social Care Outcome Tool (ASCOT) (Rand, Malley, Forder, & Netten, 2015) and the Carers’ Star (Burns, MacKeith, & Pearse, 2017).

There was no endorsement of a specific existing instrument but if there was such an instrument, the majority believed that it should be a generic carer instrument, that is, applicable to all carers. A small number disagreed and perceived difficulties in covering all relevant issues in one instrument. The majority saw potential in an instrument for identifying carers but agreed that it needed to go beyond identification to achieve benefit. Suggested domains and potential items are outlined in Table 2.

**Table 2:** Content of a carer tool

Domains	Suggested content
Caring role	Type of caring provided Amount of caring provided Support in their caring role by other informal carers Willingness and ability to continue caring role Information on person cared for o Relationship to the person o Person's condition(s) o Person's support needs
Carer needs	Unmet need in their caring role Their own health and associated needs Caring skills needed Breaks from caring
Carer outcomes	Stress or strain from caring Coping Quality of life including o Physical and mental health o Well‐being, o Ability to have a social life
Finances	Financial problems Financial support received
Employment and education	Support or ability to work or be in education

## 4 DISCUSSION

In this diverse sample of professional stakeholders, there was clear recognition of the vital importance of primary care, in collaboration with other health and care services, providing support for carers. Primary care was considered crucial in identifying and supporting carers because of its level of contact with people. It was equally clear that, although participants could cite instances of good practice, the health services generally and primary care specifically do not currently have a strong and effective role in identifying and supporting carers. Participants cited problems of time, resources and skills that previous studies have identified in primary care (Greenwood et al., 2010; Simon & Kendrick, 2001). In addition, support for the carer was seen as secondary to their more pressing role, responding to people's health problems. Strengthening the role of primary care was thought to require integrated and collaborative networks with other types of services and a cultural change towards a strengthened focus on carers. A more integrated network of services was thought to limit the demands on stretched primary care services and thus make it feasible to ensure a cultural change. There is evidence from a variety of specific sub‐groups of carers of integrated services having benefits (Ates et al., 2018; Janse, Huijsman, de Kuyper, & Fabbricotti, 2014; Lee, Yiin, & Chao, 2016; Valentini et al., 2016). However, it is difficult to generalise from these usually specialist research settings to broader contexts.

A major barrier to strengthening the role of health services in supporting carers was the basic difficulty of identifying carers, which is a long‐standing problem. Approximately 10% of the population of England are estimated to be carers but less than 1% of people are identified through general practice (Schonevegel, 2013). Few countries have a mechanism for identifying carers (Courtin et al., 2014). Our study provides evidence that little progress has been made with improving identification of carers in England and there was a distinct lack of leadership in focusing on carers. The difficulty with identification arose from two distinct sources. First, carers often fail to define themselves as carers. This is a well‐recognised problem that is becoming better understood (e.g. (Carduff et al., 2014; Carduff et al., 2016) but is still not addressed effectively. The second problem was that primary care services were seen as ambivalent about proactively identifying carers. This was thought to be because of lack of resources. There is also the possibility of facing challenging levels, types and complexity of unmet need. Some good practice examples have been given but overall it was thought that identification of carers in primary care was ad hoc and low priority. The recently published NHS plan (National Health Service (NHS), 2019) restates the commitment to carer identification, recognition and support; and aims to encourage the national adoption of carer's passports. A carer passport will identify someone as a carer and enable staff to involve the passport holder in the care of the service user (https://www.carerspassports.uk/, accessed 21.10.2019).

An area of relative optimism was the widespread view that it was feasible to assess the needs of carers. There was agreement that a standard generic approach was most appropriate, rather than using separate instruments for different long‐term chronic conditions. A carer needs assessment should adopt a holistic approach to well‐being to make it relevant to the widest range of carers and addressing physical, emotional, social and financial needs. A few respondents cited existing examples of instruments on which a carer assessment could be build such as the ASCOT‐Carer (Rand et al., 2015) or the Carer Star (Burns et al., 2017). Although not specifically cited by study participants, existing instruments such as the Carer Support Needs Assessment Tool (CSNAT) (Ewing, Brundle, Payne, & Grande, 2013) are designed to play the role envisaged by several respondents. A recent systematic review found that self‐reported carer needs questionnaires are targeted at specific sub‐population and concluded that there is no suitable validated carer needs questionnaire (Lefranc et al., 2017), which points towards the need for a new instrument to be developed.

There was widespread recognition that using a carer needs assessment instrument was only acceptable if it was likely to benefit carers. Two distinct kinds of benefits were envisaged, (a) specific benefits through support such as receipt of helpful advice, information, respite services or treatments; and (b) non‐specific benefits arising from recognition of the carer role such as communication with the carer. Feasible methods to link responses to needs assessment to specific interventions would need to be developed. Little research has been conducted on the benefits of using a carer tool but the limited evidence points towards positive impacts including, for example, an improvement in the clinician‐carer relationship or improved ability to identify appropriate support services (Guberman, Keefe, Fancey, & Barylak, 2007; Guberman et al., 2003).

It is a strength of the study that it incorporated a broad range of perspectives, but it should be acknowledged that a pragmatic sample was recruited. Although a geographical spread was achieved, this was limited to specific areas of England in particular for GP practices (Oxford, Kent, North‐West England and London as the authors had established contact with those GPs) and local councils or CCGs (predominantly Kent and Oxford). In addition, some participants entered the study via snowball sampling technique. Taken together, this sampling approach may have led to a bias towards respondents sharing similar ideas. Carers’ and service users’ views were not captured, but these would be equally important to consider in future studies. It should also be noted that the qualitative nature of this study may limit the generalisability of the findings. Other methods, such as a survey, could be used to explore the findings from this study in a large sample of professional stakeholders.

## 5 CONCLUSIONS

The health status and healthcare experiences of carers are poorer than comparable primary care users without caring responsibilities (Thomas et al., 2015). It is clear from the current study that professional stakeholders are supportive of a more proactive approach to carers through primary care, in collaboration with other health and care services, and the voluntary sector. Although many barriers to enhancing strategies for carer identification and support have been highlighted, the participants believed a primary care approach was feasible provided it resulted in benefits to carers. The uncertainties expressed by participants as to the feasibility and impact of interventions to improve carers support indicate the need for pilots and experiments to develop the evidence base. Such studies would need to be sensitive to issues of language and identity as to evidence of impact of interventions on carers’ well‐being (Larkin, Henwood, & Milne, 2019), as well as sensitive to issues of the workload and culture of primary care, and other health and care services. Given the crucial importance of carers, such studies should be a high priority.

## AUTHOR CONTRIBUTION

MP, SR and RF have conceived and designed the study. SR collected part of the data (the remainder collected by a research officer Louise Geneen – see acknowledgments). MP led on the analysis but all authors were involved in developing the analytical framework and in the interpretation of the data. All authors contributed to the writing of the manuscript, and agree to be accountable for all aspects of the work.
